# SERVICE PROVIDERS' PERSPECTIVES OF UNDERREPORTING ABUSE OF OLDER ADULTS IN ALBERTA, CANADA

**DOI:** 10.1093/geroni/igz038.3400

**Published:** 2019-11-08

**Authors:** Carla R Liepert, Christine Walsh, Kerstin Roger, Donna Goodridge, Michelle Ranville

**Affiliations:** 1 University of Calgary, Calgary, Alberta, Canada; 2 University of Manitoba, Winnipeg, Manitoba, Canada; 3 University of Saskatchewan, Saskatoon, Saskatchewan, Canada; 4 A & O: Support Services for Older Adults, Winnipeg, Manitoba, Canada

## Abstract

Minimal research has investigated disclosure/non-disclosure of abuse of older adults. To address this gap, this exploratory, qualitative study gathered the perspectives on reporting of elder abuse from 10 service providers working with elder abuse survivors across Alberta. Face-to-face and online interviews were conducted, audio-recorded, transcribed, and thematically analyzed. Four major barriers to abuse disclosure for older adults were identified. First, complex parent/adult-child relationships reduce the likelihood of disclosure among older adults who are experiencing abuse perpetrated by a family member; the corollary is that disclosure for non-familial perpetrators results in higher rates of disclosure. Second, older adults residing in rural and remote locations face greater barriers to disclosure compared to those residing in urban/suburban settings. Third, older adults are unlikely to report even if they recognize that they are in a situation of abuse, as a consequence of their internalized ageist beliefs that policing and social services agencies have more pressing needs to address than their well-being. Finally, local policing bodies and legal authorities may inadequately serve older adults facing abuse, particularly in cases of non-physical abuse, due to prioritized client and community needs. This study highlights the need for additional supports and service provision for vulnerable older adults in Alberta, specifically for those residing in rural and remote regions and those dependent on family members. It also points to the critical need for a greater understanding and awareness of violence against older adults among the general public and those tasked with ensuring the safety and well-being of older adults.

